# Use of Motor Abundance in Young and Older Adults during Dual-Task Treadmill Walking

**DOI:** 10.1371/journal.pone.0041306

**Published:** 2012-07-20

**Authors:** Leslie M. Decker, Fabien Cignetti, Jane F. Potter, Stephanie A. Studenski, Nicholas Stergiou

**Affiliations:** 1 Nebraska Biomechanics Core Facility, University of Nebraska at Omaha, Omaha, Nebraska, United States of America; 2 Laboratoire de Neurosciences Cognitives (UMR 7291), CNRS & Aix-Marseille Université, Marseille, France; 3 Division of Geriatrics, University of Nebraska Medical Center, Omaha, Nebraska, United States of America; 4 Division of Geriatric Medicine, University of Pittsburgh, Pittsburgh, Pennsylvania, United States of America; 5 College of Public Health Department, University of Nebraska Medical Center, Omaha, Nebraska, United States of America; University of Pittsburgh, United States of America

## Abstract

Motor abundance allows individuals to perform any task reliably while being variable in movement's particulars. The study investigated age-related differences in this feature when young adults (YA) and older adults (OA) performed challenging tasks, namely treadmill walking alone and while performing a cognitive task. A goal function for treadmill walking was first defined, i.e., maintain constant speed at each step, which led to a goal equivalent manifold (GEM) containing all combinations of step time and step length that equally satisfied the function. Given the GEM, amounts of goal-equivalent and non-goal-equivalent variability were afterwards determined and used to define an index providing information about the set of effective motor solutions relative to the GEM. The set was limited in OA compared to YA in treadmill walking alone, indicating that OA made less flexible use of motor abundance than YA. However, this differentiation between YA and OA disappeared when concurrently performing the cognitive task. It is proposed that OA might have benefited from cognitive compensation.

## Introduction

Walking is a complex task whose performance relies not only on the sensorimotor system but also critically depends on cognitive resources, specifically executive function that orchestrates goal-directed activities [1], [2]. A usual way to assess the extent to which gait places demands on them is to examine the individuals' ability for dual tasking, which consists in walking while simultaneously performing a secondary cognitive task. Any dual-task-related change in gait reflects that the high-order cognitive resources needed to perform the two tasks concurrently exceed the total capacity of the system so that a reduction of the resources allocated to gait performance occurs [3], [4]. Overall, studies reported an age-related dual-task deficit while walking overground, which includes larger decreased speed and increased stride-to-stride variability in older adults (OA) than in young adults (YA) [5]–[8]. This deterioration of the dual-task ability with normal aging was also observed in treadmill walking, with larger changes in the variability of the gait patterns when increasing cognitive task difficulty in OA as compared to YA [9], [10]. Moreover, the dual-task ability was found to be further deteriorated with pathological aging, for instance in case of elderly idiopathic fallers or patients suffering either from Alzheimer's disease or Parkinson's disease [11]–[13]. A likely explanation for these results is that gait control increasingly relies on cognitive processes with aging and age-related neurodegenerative diseases while at the same time attentional capacity and other relevant cognitive resources (“cognitive supply”) are reduced [14].

However, the previous studies are limited by the fact that the observed dual-task-related changes in gait parameters do not provide complete information on the extent to which taxing cognitive processes affects the individuals' walking execution. Indeed, given the number of joints and muscles in the human body, there are typically an infinite number of ways, or solution strategies, for any individual to achieve walking reliably and repetitively while allowing a relatively high variability in the movement's particulars, a feature known as equifinality [15], [16]. In the presence of equifinality, a difficulty is, therefore, to reach a conclusion as to whether dual-task-dependent gait changes reflect, or not, suboptimal solutions for completing the walking task. Consider, for example, studies that have examined changes in stride-to-stride variability during treadmill walking when increasing the difficulty of the concurrent cognitive task [9], [10]. The decreased variability observed at a low difficulty level of cognitive activity was treated as evidence of an improved gait control, while the increased variability at higher difficulty levels was treated as evidence of an altered gait control. Although such interpretations have led to elegant hypotheses with respect to the mechanisms linking gait control to cognitive demand in YA and OA, inferring beneficial and detrimental effects of cognitive activities on gait control from changes in stride-to-stride variability is tricky. Increased or decreased variability is commonly reported in populations with gait abnormalities, such as elderly fallers [17], [18] and individuals with neurodegenerative diseases (e.g. Parkinson's disease) [19], [20], so that too much or too little stride-to-stride variability may be both not optimal in terms of gait control [21]. Therefore, being able to examine dual-tasking effects on gait by answering unambiguously the question of whether or not the walking task is completed appropriately is imperative. In particular, grasping such a functional meaning would be important to make further progress in understanding the complex relationship between falls and gait changes under dual-task conditions [22]. The purpose of the present study was thus to examine age-related dual-task changes in the individuals' walking strategies approached within an equifinality-based framework.

The issue of equifinality in motor tasks has been addressed using geometry-based approaches, including those of the uncontrolled manifold [23], [24], the minimum intervention principle [25], [26], and the body-goal mapping variability [16]. These approaches share the idea that the excess of body-level degrees of freedom over those at the task-goal level gives rise to an entire set of motor solutions, assumed to have the structure of a manifold in the body's variable space. Practically, one can imagine the task of positioning its finger at a particular location in space [27]. In this example, the position of the finger is unique while many joint angle combinations (e.g., combinations of the shoulder, elbow and wrist joint angles) can be adopted to achieve that particular end-effector position. These combinations lie on a multi-dimensional surface in joint space referred to as a manifold, with any point in the manifold representing a goal-equivalent solution to the positioning task. Therefore, achieving a task with a well-prescribed goal comes then down to selecting primarily solutions within the manifold rather than outside of it. This conception can be operationalized by partitioning the variability in the body's variable space (e.g., joint space) into a component that is tangential to the manifold and a component that is orthogonal to it. The component tangential to the manifold is goal-equivalent (i.e., goal-equivalent variability) and is consistent with no variability at the level of the task variable (e.g., the finger position). Alternatively, the component orthogonal to the manifold is non-goal-equivalent (i.e., non-goal-equivalent variability) and is consistent with a variable task variable. As a result, the central nervous system preferentially constrains non-goal-equivalent variability that matter for the stabilization of the task variable and the successful achievement of the task than goal-equivalent variability that do not. With aging, previous studies on finger coordination and manual pointing reported age-related differences in the relative proportion of goal-equivalent variability, with lower ratios of goal-equivalent to non-goal-equivalent variability in OA as compared to YA [28]–[31]. The sets of effective motor solutions relative to the manifold (or the motor synergies [32]) used by OA to achieve the tasks were thus more limited than those of YA, reflecting a more conservative strategy that consists of constraining the body-level degrees of freedom (or the motor abundance [33]). Such a strategy was proposed to cope with age-related decline in sensorimotor processing [34].

Recently, a step forward in understanding how the central nervous system regulates walking on treadmill was made in the context of equifinality [35]. Since treadmill walking only requires that individuals do not walk off either the front or back end of the treadmill, a goal function was defined: maintain a constant speed at each stride, which mathematically expresses one possible control strategy. This goal function typically leads to a goal equivalent manifold (GEM) containing all the possible stride time and stride length combinations that equally satisfy the function (see section ‘*data processing and analysis’* for details). To determine whether humans adopt a strategy that recognizes the GEM, the authors quantified tangential and perpendicular deviations from the GEM [35]. The former deviations are goal-equivalent because they do not affect walking speed while the latter deviations that are non-goal-equivalent do. Goal-equivalent deviations were found to be more widely dispersed than non-goal-equivalent deviations, meaning that humans minimize errors relative to the GEM. In addition, humans immediately corrected non-goal-equivalent deviations at each successive stride, while allowing goal-equivalent deviations to persist across multiple strides. Taken together, these results clearly indicated that humans exploit motor abundance, and so equifinality, to control walking on treadmill.

The present study is built on the analytical framework of a GEM-based control of gait, with YA and OA participating in single- and dual-task treadmill walking. In dual-task, the concurrent cognitive task to treadmill walking was the Boston Naming Test [36]. This test consists in a series of pictures of objects (ranging from high frequency to rare object names) that the participant is required to name. Confrontation naming activates regions involved in executive and word retrieval processes [37]. Thus, naming pictured objects (as found in the Boston Naming Test, employed in the current design) consumes cognitive resources and is likely to cause a mismatch between the resources available and those required for treadmill walking under a dual-task condition. In light of age-related changes in motor synergies, a first hypothesis was that OA would show a lower relative proportion of variability along the GEM (i.e., a more limited set of effective motor solutions relative to GEM) than YA in single-task treadmill walking. Based on age-related dual-task deficit, a second hypothesis was that dual tasking would magnify the previous differentiation between OA and YA (i.e., a *group*×*task* interaction effect), involving an even more pronounced reduction of the relative proportion of variability along the GEM (i.e., a much more limited set of effective motor solutions relative to GEM) in OA as compared to YA.

## Methods

### Participants

YA (*n* = 23) aged between 20 and 35 years and OA (*n* = 19) aged at least 65 years were recruited for the experiment. The two samples matched regarding their demographic characteristics (Table 1). All participants were: (i) right-handed; (ii) living independently in the community; (iii) able to ambulate without the use of, or assistance from, a prosthetic device, a fixed or mobile walking frame, or other assistive devices (e.g., brace, cane, crutch), or without the assistance of another person; and (iv) not diagnosed with neurologic conditions (e.g., Alzheimer's disease, Parkinson's disease, stroke, and multiple sclerosis) or other conditions (e.g., dementia, moderate or severe chronic obstructive pulmonary disease, weight-bearing pain, chest pain at rest or during activity, previous history of myocardial infarction, dyspnea at rest or use of supplemental oxygen) that may impair the participant's safety during the procedures outlined in the protocol. Informed consent was obtained from all participants prior to data collection according to the guidelines of the University's Institutional Review Board.

**Table 1:** Demographic characteristics of the participants.

	Younger	Older	Statistics
Gender (M/F)	11/12	12/7	*x* ^2^(1, *N* = 42) = 0.99; *p* = .32
Age (years)	23.56±0.69	70.95±1.47	***t*** **(40) = −30.83; ** ***p*** **<.0001**
Height (m)	1.75±0.02	1.71±0.02	*t*(40) = 1.19; *p* = .26
Weight (kg)	71.12±2.88	77.69±3.09	*t*(40) = −1.54; *p* = .12
Education (years)	16.35±0.4	17.26±0.81	*t*(40) = −1.06; *p* = .29

Entries are mean ± standard error.

### Experimental procedure and data collection

The participants first underwent tests of general cognitive functioning, involving the Mini Mental State Exam [38]–[40] and the Wechsler Adult Intelligence Scale-3^rd^ Edition (WAIS-III) forward and backward digit span tests [41], [42]. Vocabulary ability was also determined using the vocabulary subtest of the WAIS-III, which was required for interpreting correctly scores on the Boston Naming Test as explained afterwards. Further, baseline data with respect to the number of falls in the year prior to the experiment, fear of falling (Modified Falls Efficacy Scale [43]) and depression (15-item Geriatric Depression Scale [44]) were obtained in OA.

The experiment then involved a sitting session and a walking session. In the former session, participants performed the Boston Naming Test while being seated to establish the baseline cognitive performance. In the latter session, participants walked at their preferred walking speed (PWS) on a treadmill with and without simultaneous confrontation naming performance. The two experimental sessions were counterbalanced across participants to account for possible order effects.

The sitting session consisted in establishing a baseline for confrontation naming performance. Participants were asked to name aloud a series of pictures of objects displayed on a screen at a fixed rate of four seconds per item. The confrontation naming test lasted three minutes; hence, 45 items were presented successively to participants. The items ranged from common ones like ‘pencil’ or ‘tree’ to less familiar ones like ‘sphinx’ and were randomly taken from the 60-item Boston Naming Test [36]. Despite its apparent simplicity, the confrontation naming task recruit a complex set of mental representations and cognitive processes, notably: (i) recognition of the visual stimulus, (ii) access of the meaning of the pictured object in the semantic system, (iii) retrieval of the lexical representation and access of its phonological word form in the lexical system, and (iv) planning of the motor programs that drive articulation [45]. Thus, this task activates regions involved in executive processes [37], and is commonly employed by neuropsychologists to assess executive functioning [46], [47].

The walking session consisted first in attaching reflective markers to a tight fitting suit at specific anatomical landmarks of each participant's lower limbs [48]–[49]. The anatomical landmarks were the anterior and posterior superior iliac spine, lumbosacral joint, greater trochanter of the femur, lateral mid-thigh, front lower thigh, lateral and medial epicondyles of the femur, front mid-shank, lateral lower shank, lateral and medial malleoli, lateral border of the fifth metatarsal head, medial border of the first metatarsal head, lateral and medial processes of the calcaneal tuberosity, heel, and between the second and third metatarsophalangeal joints. Participants were then given ample time to familiarize themselves with treadmill walking. The PWS was established using a well-established protocol [50]. Initially, the participants walked at a relatively slow speed, and then the investigator increased the speed in 0.1 km.h^−1^ increments until the participants reported their PWS. The speed was then increased by approximately 1.5 km.h^−1^ and decreased by 0.1 km.h^−1^ until the PWS was re-established. This procedure was repeated until a close match was achieved (less than 0.4 km.h^−1^ difference). Afterwards, participants walked under a single-task (control) condition (i.e., walking without an explicit cognitive requirement) and a dual-task condition (i.e., walking while simultaneously performing the confrontation naming task as implemented in the sitting session). Each condition was conducted at PWS and lasted for three minutes. This duration was chosen because it was difficult for the participants, especially OA, to sustain attention for a longer period of time. Ample rest time was provided between the two conditions. The three-dimensional positions of the markers were collected at 60 Hz with an eight high-speed cameras Motion Analysis Eagle Digital system using EVaRT software (version 5.0, Motion Analysis Corporation, Santa Rosa, CA).

### Data processing and analysis

**Figure 1:**
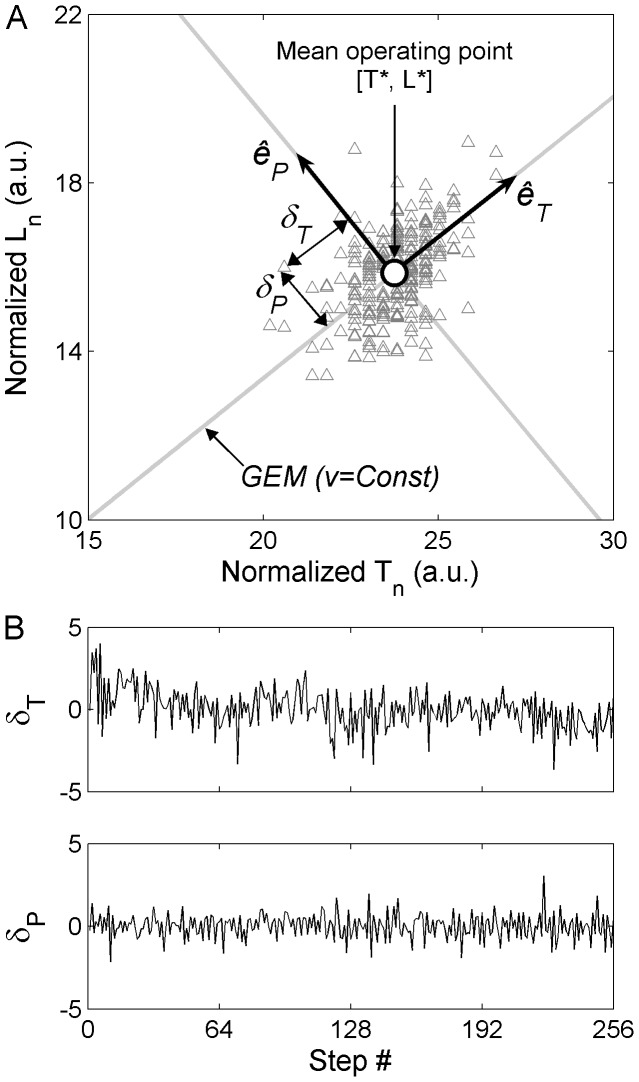
Goal Equivalent Manifold (GEM) analysis for treadmill walking. (**A**) Step length (*L_n_*) and step time (*T_n_*) were first normalized to unit variance to facilitate the analysis. A triangle represents a particular [*L_n_, T_n_*] combination for one individual step. The GEM was then defined from all [*L_n_*, *T_n_*] combinations that achieved the exact same speed *v* as defined by the goal function (cf. Eq. 2), and was a diagonal solid line. The triangles thus represent the set of effective motor solution relative to the GEM used to achieve treadmill walking. Next, an operating point [*T* ^*^, *L* ^*^] and orthonormal basis vectors 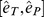 centered on this point and aligned tangent to and perpendicular to the GEM were defined. (**B**) Finally, deviations along the GEM, 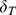, and perpendicular to the GEM, 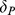, were obtained using a linear coordinate transformation (cf. Eq. 3). The relative proportion of variability along the GEM was evaluated from the ratio 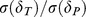. The lower the ratio, the lower the relative amount of variability along the GEM, and the more limited the set of effective motor solutions relative to the GEM.

Confrontation naming was scored based on the percentage of correct responses [37], [51], with a correct response occurring when a picture was identified properly. With respect to gait data, the marker trajectories were first low-pass filtered at 10 Hz with a zero-lag Butterworth filter. Data were then analyzed in the framework of the GEM for treadmill walking [35]. The primary requirement for treadmill walking with speed *v* is to not walk off the treadmill. Since the net change in displacement for step *n* is a function of step length, *L_n_*, and step time, *T_n_*, as *L_n_* – *vT_n_*, treadmill walking was formulated as:
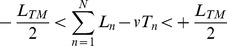
(1)where the summation is the net displacement walked over *N* steps and *L_TM_* is the treadmill length. The simplest strategy to satisfy Eq. 1, and then to perform the walking task, is to keep *v* constant at each step, which was formulated using the goal function:
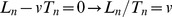
(2)All [*L_n_*, *T_n_*] pairs that satisfied Eq. 2 defined the GEM, which was typically a solid line in the [*L_n_*, *T_n_*] plane (Fig. 1A). *T_n_* and *L_n_* were obtained from the time interval and horizontal distance between consecutive toe-off events, respectively. The toe-off was defined as the maximum backward displacement of the toe marker (i.e., the marker located between the second and third metatarsophalangeal joints) during each step. For consistency across subjects, both *L_n_* and *T_n_* time series were shortened to *n* = 256 steps, which was the number of steps of the slowest subject. Each series was also normalized to unit variance by dividing it by its own standard deviation, which provided an intuitive reference for comparison between participants and conditions [29]. The velocity for step *n* was subsequently defined as *S_n_* = *L_n_*/*T_n_* and the average walking speed was obtained as 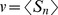, where 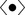
denotes the average over the 256 steps.

Next, an operating point of coordinates 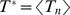 and 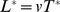 and orthonormal basis vectors 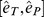 centered on this point and aligned tangent to and perpendicular to the GEM were defined (Fig. 1A). The coordinates were then re-expressed as 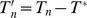 and 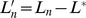. Finally, (goal-equivalent) deviations along the GEM, 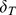, and (non-goal-equivalent) deviations perpendicular to the GEM, 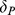, were calculated as follows (Fig. 1B):
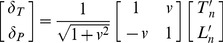
(3)Standard deviations (σ) for 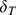 and 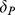 time series were computed and the relative proportion of variability along the GEM was calculated as the ratio 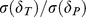. Considering that 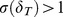 and 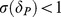, higher variability is present along the GEM than perpendicular to it [35]. It reflects that humans adopt a strategy that recognizes the GEM. The lower the ratio, the lower the relative proportion of variability along the GEM and the more limited the set of effective motor solutions relative to the GEM.

Scaling exponents *α* were also computed from 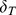 and 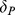 time series using the Detrended Fluctuation Analysis [52]–[54]. This provided information about the rapidity of the participants to correct deviations along and perpendicular to the GEM. Briefly, the mean square roots of (linearly) detrended residuals, *F*(*n*), of the integrated 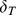 and 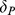 time series were calculated over a range of box lengths *n*, with *n* the number of steps. The *log_10_*[*F*(*n*)] vs. *log_10_*(*n*) plots were then fitted with a linear function and *α* was obtained from the slope of this line over the range of box lengths *n* = 17 to *n* = 45. This range provided the most stable estimates of *α*, as determined using a DFBETA procedure [55]. When *α*<0.5, the time series contained anti-persistent correlations, consistent with an immediate correction to the GEM. When *α*>0.5, the time series contained persistent correlations, reflecting a correction to the GEM that is not immediate [35], [56]. All gait data were processed using MATLAB (Mathworks, Natick, MA, USA).

### Statistical analysis

The Kolmogorov-Smirnov test confirmed the normality of distribution for all dependent variables (i.e., % correct responses for confrontation naming, and *σ* and *α* for gait). General Linear Models for analyses of variances (ANOVAs) with between- and within-subjects factors were subsequently used. The factors were *group* (YA *vs.* OA) and *session* (sitting *vs.* walking) for performance in confrontation naming, and *group* and *task* (single-task walking *vs.* dual-task walking) for gait. Importantly, ANOVA results for confrontation naming and gait were adjusted for the covariates vocabulary and velocity, respectively. The former adjustment accounted for false-positive rates for naming deficit due to poorer vocabulary knowledge [57], [58], and the latter adjustment for gait differences resulting from differences in gait velocity [50], [59]–[61]. Adjusted (least squares) mean (*M*) and standard error of the mean (*SE*) are then reported for all dependent variables. When ANOVAs yielded significant results, post-hoc multiple comparisons were conducted using the Tukey's HSD test to examine for differences between the factors' levels. Effect sizes are reported as *η* ^2^ = SS_explained_/SS_total_. Statistical significance was set at 0.05. Statistica *v*10 (Statsoft, Inc., Tulsa, OK, USA) was used to perform all analyses.

## Results

### Baseline characteristics of the YA and OA

Baseline characteristics of the analytic samples are shown in Table 2. All participants scored more than 25 on the Mini Mental State Exam, and more than 6 and 5 on the WAIS-III forward and backward digit span tasks, respectively, which reflected a preserved general cognitive function and working memory. Moreover, OA reported no falls in the year prior to the experiment, had no fear of falling (i.e., scores close to 10 on the Modified Falls Efficacy Scale), and had an average score of 1 on the 15-item Geriatric Depression Scale that indicated no symptoms of depression. Finally, vocabulary performance and preferred walking speed were significantly higher and lower in OA as compared to YA. As previously mentioned, such differences were statistically controlled in the ANOVAs by co-varying vocabulary and speed to avoid confounding.

**Table 2:** Baseline characteristics of the participants.

			Younger	Older	Statistics
**Cognition**	Digit Span	*Forw.*	11.6±0.54	10.68±0.46	*t*(40) = 1.26; *p* = .21
		*Back.*	7.74±0.45	6.47±0.55	*t*(40) = 1.77; *p* = .08
		*Tot.*	19.35±0.88	17.21±0.88	*t*(40) = 1.69; *p* = .09
	MMSE		29±0.21	28.89±0.31	*t*(40) = 0.28; *p* = .77
**Depression**	GDS			1±0.28	
**Fear of falling**	MFES			9.91±0.05	
**Vocabulary**	WAIS III		1.11±0.04	1.55±0.06	***t*** **(40) = −5.98; ** ***p*** **<.0001**
**Gait**	PWS (m/s)		1.08±0.04	0.77±0.04	***t*** **(40) = 4.86; ** ***p*** **<.0001**

Entries are mean ± standard error. MMSE: Mini Mental State Exam. GDS: Geriatric Depression Scale. MFES: Modified Falls Efficacy Scale. PWS: Preferred Walking Speed. WAIS III: Wechsler Adult Intelligence Scale-3^rd^ Edition.

### Percentage of correct responses in confrontation naming

A significant main effect of *group* (*F* [1], [39] = 5.93, *p* = 0.019, *η* ^2^ = 0.12) and a significant *group*×*session* interaction effect (*F* [1], [39] = 5.64, *p* = 0.022, *η* ^2^ = 0.14) were observed. There was no significant main effect of *session* (i.e., sitting *vs.* walking). Post-hoc examination of the interaction effect indicated that the percentage of correct responses in OA (*M* = 90.03, *SE* = 1.58) was significantly lower (*p* = 0.005) than that in YA (*M* = 97.16, *SE* = 1.48) when sitting. This percentage significantly increased (*p* = 0.02) in OA when walking (*M* = 92.84, *SE* = 1.31), but remained the same in YA (*M* = 96.03, *SE* = 1.22). Thus, OA performed worse on confrontation naming than YA when sitting, but this difference disappeared when walking as a result of an improved performance in OA.

### Variability along and perpendicular to the GEM

**Figure 2:**
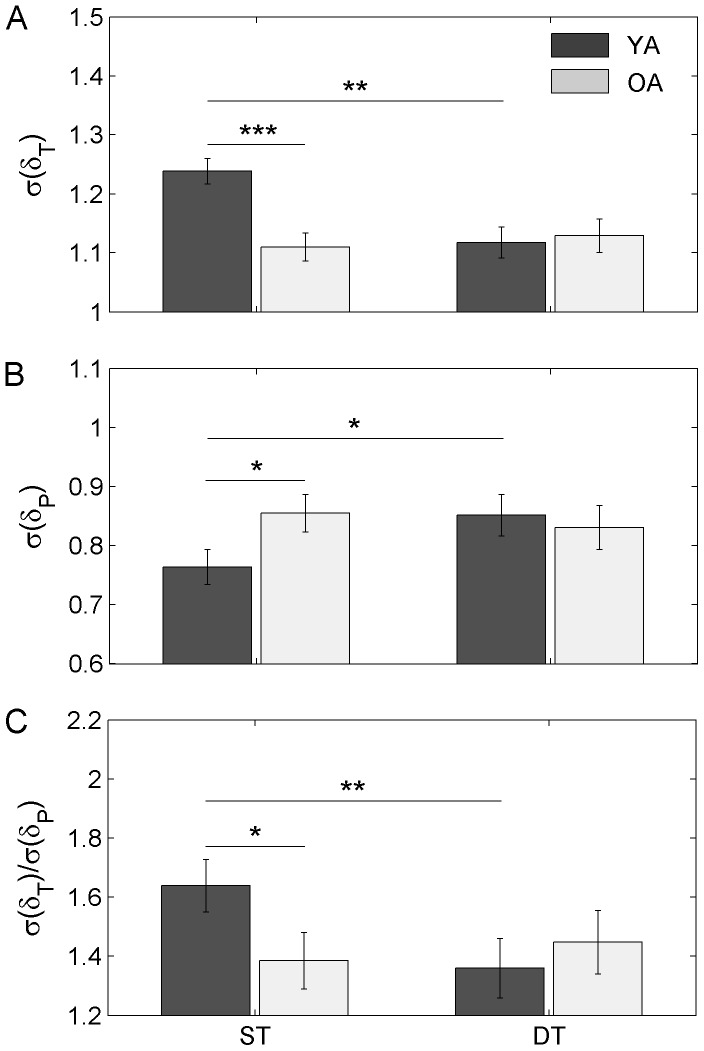
Results of the Goal Equivalent Manifold (GEM) analysis for treadmill walking in single-task (ST) and dual-task (DT). (**A**) Variability along the GEM, as evaluated from 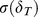. (**B**) Variability perpendicular to the GEM, as evaluated from 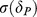. (**C**) Relative proportion of variability along the GEM, as evaluated from the ratio 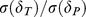. YA: Young Adults. OA: Older Adults. Significant differences are indicated by stars (^*^ *p*<0.05, ^**^ *p*<0.01, ^***^ *p*<0.001). Error bars represent standard error of the mean.

A significant main effect of *task* for 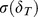, *F* [1], [38] = 12.06, *p* = 0.001, *η* ^2^ = 0.04, and 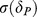, *F* [1], [38] = 8.31, *p* = 0.006, *η* ^2^ = 0.03, was observed. In addition, a *group*×*task* interaction effect was also significant for these two variables, with *F* [1], [38] = 19.47, *p*<10^−5^, *η* ^2^ = 0.07, for 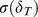, and *F* [1], [38] = 7.82, *p* = 0.008, *η* ^2^ = 0.03, for 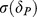. There was no significant main effect of *group*. Post-hoc tests revealed the origins of the interaction effect. First, 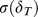 and 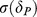 values in OA (*M* = 1.11, *SE* = 0.02 and *M* = 0.85, *SE* = 0.03; respectively) were significantly lower (*p* = 0.001) and larger (*p* = 0.024), respectively, than those in YA (*M* = 1.23, *SE* = 0.02 and *M* = 0.76, *SE* = 0.02; respectively) in the single-task condition (walking alone) (Fig. 2A, 2B). Second, significantly decreased (*p* = 0.0001) and increased (*p* = 0.01) values of 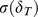 and 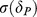, respectively, occurred for YA in the dual-task condition (*M* = 1.11, *SE* = 0.02 and *M* = 0.85, *SE* = 0.03; respectively), while no changes occurred for OA (*M* = 1.12, *SE* = 0.02 and *M* = 0.83, *SE* = 0.03; respectively) (Fig. 2A, 2B). Therefore, the GEM-based control of treadmill walking was more extensive in YA than in OA in the single-task condition, involving more variability (elongation) along the GEM (i.e., in the goal-equivalent direction) and less variability (compression) perpendicular to the GEM (i.e., in the non-goal-equivalent direction). In addition, only YA exhibited dual-task-related changes in their gait control strategy, with variability values in the goal-equivalent and non-goal-equivalent directions of the GEM decreasing and increasing, respectively, and becoming similar to those of OA.

### Relative proportion of variability along the GEM

A significant main effect of *task*, *F* [1], [38] = 5.52, *p* = 0.024, *η* ^2^ = 0.02, and a *group*×*task* interaction effect with *F* [1], [38] = 9.33, *p* = 0.004, *η* ^2^ = 0.04, were observed for the ratio 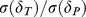. There was no significant main effect of *group*. The interaction effect comes from a statistically lower (*p* = 0.015) ratio in OA compared to YA in the single-task condition (*M* = 1.38, *SE* = 0.09 *vs. M* = 1.63, *SE* = 0.08; respectively), and a significantly reduced (*p* = 0.001) ratio in YA (*M* = 1.35, *SE* = 0.11), but not in OA (*M* = 1.44, *SE* = 0.11), in the dual-task condition (Fig. 2C). Thus, although the set of effective motor solutions relative to the GEM used by YA during single-task treadmill walking was larger than that of OA, dual tasking annihilated such a difference by making the YA's set smaller.

### Corrected deviations along and perpendicular to the GEM

**Figure 3:**
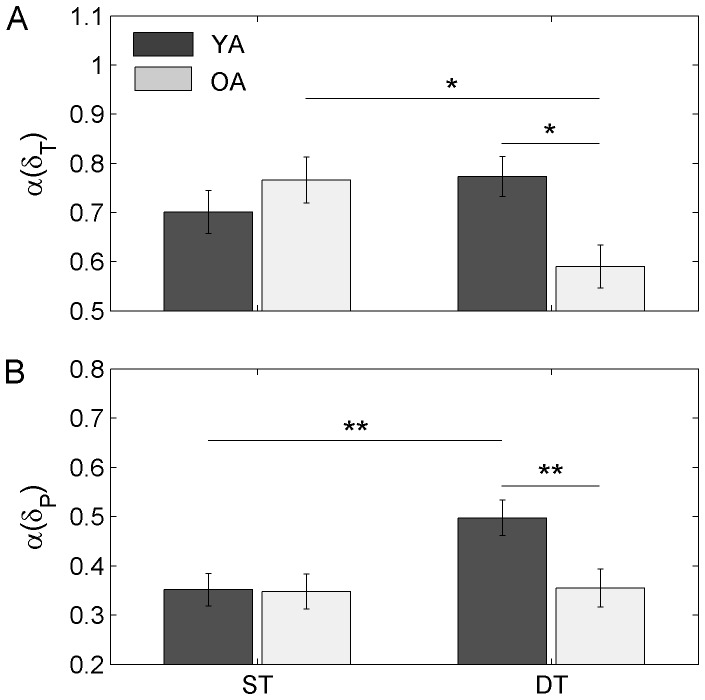
Results of the Detrended Fluctuation Analysis applied to deviations from Goal Equivalent Manifold (GEM) in single-task (ST) and dual-task (DT). (**A**) Scaling exponent of the deviations along the GEM, 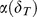. (**B**) Scaling exponent of the deviations perpendicular to the GEM, 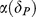. YA: Young Adults. OA: Older Adults. Significant differences are indicated by stars (^*^ *p*<0.05, ^**^ *p*<0.01). Error bars represent standard error of the mean.

A significant main effect of *task* for 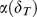, *F* [1], [38] = 3.93, *p* = 0.047, *η* ^2^ = 0.04, and 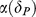, *F* [1], [38] = 5.21, *p* = 0.028, *η* ^2^ = 0.08, and a significant *group*×*task* interaction effect for 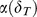, *F* [1], [38] = 6.86, *p* = 0.012, *η* ^2^ = 0.07, and 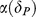, *F* [1], [38] = 6.69, *p* = 0.013, *η* ^2^ = 0.03, were observed. The interaction effect for 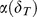 resulted from a significant decrease (*p* = 0.019) of the OA's exponent from single- to dual-task condition (*M* = 0.76, *SE* = 0.04 *vs. M* = 0.59, *SE* = 0.04; respectively) while the YA's exponent remained equivalent (*M* = 0.71, *SE* = 0.04 *vs. M* = 0.77, *SE* = 0.04; respectively); the OA's exponent being significantly lower (*p* = 0.027) than the YA's exponent in the dual-task condition (Fig. 3A). Inversely, the interaction effect for 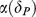 resulted from a significant increase (*p* = 0.003) of the YA's exponent from single- to dual-task condition (*M* = 0.35, *SE* = 0.03 *vs. M* = 0.49, *SE* = 0.03; respectively) while the OA's exponent remained unchanged (*M* = 0.34, *SE* = 0.03 *vs. M* = 0.35, *SE* = 0.03; respectively); the YA's exponent being significantly higher (*p* = 0.001) than the OA's exponent in the dual-task condition (Fig. 3B). Thus, these results indicated that deviations along and perpendicular to the GEM tended to become no longer corrected in OA and in YA, respectively, in the dual-task condition.

## Discussion

### Age-related differences in single-task treadmill walking

During single-task treadmill walking, OA exhibited a lower relative proportion of variability along the GEM (i.e., a lower 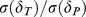 ratio) than YA, due to lower 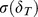 and larger 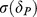. This result confirmed our hypothesis: the body-level degrees of freedom were more constrained in OA than in YA, giving rise to a more limited set of effective motor solutions relative to the GEM for successful treadmill walking. This finding echoes previous evidence that OA make less flexible use of motor abundance than YA, as revealed by examining finger coordination and manual pointing using the uncontrolled manifold method [28]–[31]. Two explanations may account for the observed age-related differences in the way body-level degrees of freedom were functionally constrained to accomplish treadmill walking. A first explanation [31], [34] stipulates that constraining motor abundance is an adaptive “choice” strategy used by OA to successfully cope with the task goal despite a system faced with sensorimotor decline, involving especially altered sensorimotor processing in the brain [62], increased neuromuscular noise [63], [64], and loss of strength and flexibility [65]. Considering that gait relies on many elements of the neuromuscular system [66]–[70], it is likely that OA were more vulnerable to unsecured gait on treadmill than YA and operated closer to their performance limits. Consequently, they might have opted for a more conservative strategy of constraining motor abundance. Moreover, it is plausible that the important affective dimension of treadmill walking (risk-taking) might have reinforced the above strategy, further restraining motor abundance in OA to ensure a safe gait. On the other hand, a second explanation [31] is that the generalized spread of (non-selective recruitment of) brain activity that occurs with normal aging when performing motor tasks, so-called dedifferentiation [14], [71], [72], would underlie the restriction of motor abundance in OA. The dedifferentiation hypothesis in fact stipulates that there is a reduced specificity of the functional cortical networks with aging so that sensorimotor representations in the central nervous system are more easily confused, which might have led at the behavioral level to less numerous effective motor solutions to cope with the treadmill walking task.

Although the explanations of an adaptive “choice” strategy or a dedifferentiation for the restricted use of motor abundance in OA are not mutually exclusive, further results from the present study rather support the former explanation. As dedifferentiation commonly leads to impairments in motor performance due to a loss of neural specialization [73], the control law for treadmill walking would likely have been altered in OA. However, no difference was found between OA and YA regarding the regulation over time of the deviations relative to the GEM, immediately correcting non-goal-equivalent deviations at each successive step (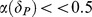) and slowly correcting goal-equivalent deviations across multiple steps (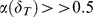). Therefore, OA may have chosen to use a more limited set of motor solutions to ensure success and safety of their gait while regulating treadmill walking in the same way YA do, by over-correcting small deviations in walking speed at each step [35]. However, future studies are warranted to investigate whether the less flexible use of motor abundance in OA reflects an adaptive strategy to account for sensorimotor decline or rather results, in a mechanistic sense, from an inability to master body-level degrees of freedom due to sensorimotor decline.

### Age-related similarities in dual-task treadmill walking

Contrary to our hypothesis, the difference between YA and OA in terms of 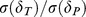 ratio was vanished, and not magnified, under the dual-task condition. This effect resulted from a decreased YA's ratio that became equivalent to that of OA when dual tasking, through a drop in 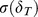 and an increase in 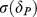. Hence, the concurrent cognitive task, namely confrontation naming, affected the arrangement of the body-level degrees of freedom only in YA in such a way that the set of effective motor solutions relative to the GEM used for treadmill walking was reduced. Considering that the most common finding in dual-task treadmill walking studies is an impaired walking performance when the two tasks are performed concurrently due to resource sharing [5]–[10], the absence of dual-task effect in OA is counterintuitive. Indeed, resources allocated to treadmill walking is reduced when performing confrontation naming, and so a more conservative strategy, that is constraining motor abundance, should have been adopted to secure gait, as observed in YA. An explanation, inspired from previous discussions about changes in effective motor solutions relative to the manifold [32], [74], may be that the lower ratio in OA in the single-task walking condition left less room for change in the dual-task condition, with OA operating already very close to their walking performance limits. Besides, changes in the way deviations in both directions of the GEM were regulated in the dual-task condition further support this explanation. Deviations from the GEM became less regulated (*α*-value closer to 0.5) in OA only in the goal-equivalent direction, i.e. in the direction in which changes do not affect walking speed and then do not challenge the goal function of treadmill walking. Inversely, deviations from the GEM became less regulated in YA only in the non-goal-equivalent direction, challenging the task goal through less controlled changes in walking speed across steps. Contrary to YA, OA thus maintained a very robust GEM-based strategy to stabilize treadmill walking performance when dual tasking, likely reflecting a lower performance limit in the sense of poorer gait flexibility and adaptability.

However, two elements call for an additional explanation. First, a control of treadmill walking that recognizes GEM occurs from the moment that 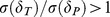 [35]. There was then theoretically room for dual-task-related gait changes as the average OA ratio in the single-task condition was 1.38±0.09. Second, OA slightly increased their performance in confrontation naming in the dual-task condition, reaching a performance level similar to that of YA. Taken together, these results suggest that OA benefited, to some extent at least, from dual tasking while YA did not. The compensation hypothesis for neurocognitive aging assumes that, under certain circumstances, networks in the aging brain can be overactivated (“work harder”) but also additional networks can be recruited to make up for neural and behavioral deficits [73], [75], allowing OA to perform equivalently to YA. Specifically, such a compensatory brain activity was evidenced, when performing complex, either cognitive [76], [77] or motor [73], tasks and occurred mainly in frontal brain regions. In the present study, older participants may have perceived the dual task sufficiently challenging to strongly rely on compensation to meet and maintain successful performance of each task at hand, gait and confrontation naming. Although such interpretation has merit, caution is required due to some limitations of the study. First, it would have been important to manipulate the dual-task demands (e.g., by increasing either the confrontation naming demand or gait demand or both) to provide strong support to the previous interpretation. If OA actually engage more neural resources for a given dual-task demand level than do YA, they would have likely reached the limit of available resources with increased dual-task difficulty, resulting in altered gait and cognitive performances. Second, the participants performed near ceiling level in naming accuracy (means ranging from 90 to 97% of correct answers), especially YA. Accordingly, the age-differential effect of dual-tasking on naming accuracy, with an improved accuracy occurring only in OA, is difficult to interpret as it might simply result from a lack of sensitivity of the measure to detect the improvement conferred by the dual task in YA. In particular, there is a body of literature that demonstrated improvement in cognitive function in both YA and OA when performing acute exercise [78], [79], as a bout of moderate treadmill walking. Accordingly, more informative measures of cognitive function would have been worth to be considered (e.g., reaction time during confrontation naming) to test further the hypothesis of cognitive compensation.

In conclusion, findings from the present study are twofold. First, the relative proportion of variability along the GEM was smaller in OA compared to YA under single-task treadmill walking. This result revealed that the former makes a less flexible use of motor abundance than the latter and extended previous findings obtained with simpler motor tasks. Second, the differentiation between OA and YA in the use of motor abundance disappeared under the dual-task condition, with a drop in the relative proportion of variability along the GEM occurring only in YA. Furthermore, performance of OA in the concurrent cognitive (confrontation naming) task to treadmill walking appeared to slightly increased, reaching a level similar to that of YA. Thus, gait and cognitive performance of OA benefited from dual tasking to some extent. An explanation might be that when OA attempt to perform two tasks at once, such as walking and confrontation naming, they may strongly rely on neural compensation to stabilize both task performance. Future studies are needed to test such compensation hypothesis and to evaluate whether previous results extend to other samples of less vigorous OA, as frail OA and OA with cognitive impairments or high risk of falling. This latter point would contribute better characterizing these phenotypes.
